# Outcomes of Pemetrexed-based chemotherapies in *HER2*-mutant lung cancers

**DOI:** 10.1186/s12885-018-4277-x

**Published:** 2018-03-27

**Authors:** Yan Wang, Shijia Zhang, Fengying Wu, Jing Zhao, Xuefei Li, Chao Zhao, Shengxiang Ren, Caicun Zhou

**Affiliations:** 1 Department of Medical Oncology, Shanghai Pulmonary Hospital, Tongji University School of Medicine, Tongji University Medical School Cancer Institute, No. 507 Zheng Min Road, Shanghai, 200433 People’s Republic of China; 2 Department of Lung Cancer and Immunology, Shanghai Pulmonary Hospital, Tongji University School of Medicine, Shanghai, People’s Republic of China; 3 Department of Respiratory Medicine, Huaihe Hospital, Henan University, Kaifeng, People’s Republic of China

**Keywords:** *HER2* mutation, Lung adenocarcinoma, Pemetrexed

## Abstract

**Background:**

*HER2* mutation has been found to be an oncogenic driver gene in non-small cell lung cancers(NSCLC) and HER2-directed therapies have shown promising results in this unique population, while little is known about its association with outcomes of chemotherapy. The aim of this study was to investigate the efficacy of first line chemotherapy in patients with advanced *HER2*-mutant lung adenocarcinomas.

**Methods:**

Patients with advanced NSCLC(*N* = 1714) initially underwent testing for *EGFR*, *KRAS*, *BRAF* mutations and *ALK*, *ROS1* rearrangements, and negative cases were then assessed for *HER2* mutations using the method of amplification refractory mutation system(ARMS). The efficacy of first line pemetrexed-based chemotherapy was investigated in patients with *HER2*-mutant and those with *EGFR*-mutant, *ALK*/*ROS1*-rearranged and *KRAS*-mutant advanced adenocarcinomas.

**Results:**

*HER2* mutations were detected in 29 of 572(5.1%) specimens from a selected population of *EGFR*/*KRAS*/*BRAF*/*ALK*/*ROS1* negative patients. All of them are adenocarcinomas. Among patients with *HER2*-mutant lung cancers, 25 received pemetrexed-based first line chemotherapy. The objective response rate(ORR) was 36.0%. Their median progression free survival(PFS) was 5.1 months, which was similar with that of *KRAS*-mutant group (*n* = 40,5.0 months, *p* = 0.971), numerically shorter than that of *EGFR*-mutant group(*n* = 74, 6.5 months, *p* = 0.247) and statistically significantly shorter than that of *ALK*/*ROS1*-rearranged group (*n* = 39,9.2 months, *p* = 0.004). Furthermore, *HER2* variants subgroup analysis showed that PFS was inferior in A775_G776insYVMA group compared with other variants (4.2 vs 7.2 months, *p* = 0.085).

**Conclusions:**

Patients with advanced *HER2*-mutant lung adenocarcinomas showed an inferior outcome of first line pemetrexed-based chemotherapy compared to those with *ALK*/*ROS1* rearrangements, which strengthen the need for effective HER2-targeted drugs in clinical practice.

**Electronic supplementary material:**

The online version of this article (10.1186/s12885-018-4277-x) contains supplementary material, which is available to authorized users.

## Background

Human epidermal growth factor receptor2(*HER2*) positivity is well-studied in breast cancer, while much less defined in lung cancer. Although anti-HER2 monoclonal antibody such as trastuzumab has been proven effective in breast cancer and gastric cancer [1, 2], the clinical trials [3, 4] of lung cancer including patients treated with trastuzumab combined with chemotherapy failed to demonstrate benefit in survival in HER2 IHC positive patients. Besides that, pan-HER TKI dacomitinib also showed no response in patients with HER2 amplifications in a phase II trial [5].

Apart from HER2 over-expression and amplification, *HER2* gene mutation is a distinct entity in lung carcinogenesis with an incidence of 4.8% among *EGFR* wild-type lung adenocarcinoma resection samples [6]. Drugs that target *HER2* gene mutations are currently being investigated. The National Comprehensive Cancer Network (NCCN) recommend trastuzumab or afatinib as potential therapy options for non-small cell lung cancers(NSCLC) patients with *HER2* mutations. Several phase I/II trials [5, 7–9] is now investigating the efficacy of other irreversible pan-HER receptor family inhibitors, such as dacomitinib, neratinib and pyrotinib. Currently, *HER2* mutation is emerging as a promising druggable target, while the optimal choice of targeted therapy remains poorly defined.

Chemotherapy is still the standard first-line regimen for patients with advanced NSCLC who are improper for targeted therapy. Among them, pemetrexed-based regimen has showed superior efficacy with less side effects and was recommended preferentially for patients with adenocarcinomas [10, 11]. *ALK*/*ROS1*/*RET* positive patients showed a superior progression free survival(PFS) after pemetrexed-based therapy than patients with *KRAS* mutations [12–16]. While the effects of *HER2* mutation on the outcomes of pemetrexed-based chemotherapy is still unknown in patients with advanced NSCLC.

Aim to investigate the efficacy of pemetrexed-based chemotherapy in patients with *HER2*-mutant lung adenocarcinomas, we conducted this retrospective study in Chinese patients with 1714 advanced NSCLC. In addition, we also observed the clinicopathologic and molecular features of *HER2* mutations in patients with advanced NSCLC.

## Methods

### Patients population

Patients with advanced NSCLC (stage IIIB/IV) and performed *EGFR*, *ALK*, *ROS1*, *BRAF* and *KRAS* testing at Shanghai Pulmonary Hospital, Tongji University School of Medicine, Shanghai, China from January 2015 to September 2016 were included into this study. *HER2* mutations testing were performed in all these 5 genes pan-negative patients. Their clinical data were collected including age, gender, smoking status, tumor histology, performance status (PS) and the outcomes of anti-cancer therapies. Patients with *HER2*, *EGFR* or *KRAS* mutation or *ALK* or *ROS1* rearrangement and received first-line pemetrexed-based chemotherapy (pemetrexed monotherapy or combination therapy with platinum) were eligible for analysis. A history of radiotherapy, first-line targeted therapy, or immune-directed therapy was exclusionary.

### Molecular testing

*HER2* mutation testing was performed using the method of amplification refractory mutation system(ARMS) by ADx HER2 Mutation Detection Kit (Amoy Diagnostics, Xiamen, China). Samples positive for *HER2* mutations were confirmed by DNA sequencing using primers with the following sequences: 5’GCC ATG GCT GTG GTT TGT GAT AGG3’ (forward) and 5’ATC CTA GCC CCT TGT GGA CAT AGG3’, which amplified a 342-bp fragment in exon20 of the *HER2* gene. The details can be referred to our previous study [6].

Similarly, *EGFR*, *BRAF* and *KRAS* mutation were performed using EGFR, BRAF V600 and KRAS Mutations Detection Kit (Amoy Diagnostics, Xiamen, China) respectively by ARMS method. *ALK* and *ROS1* rearrangement testing were performed using AmoyDx EML4-ALK and ROS1 Fusions Detection Kit (Amoy Diagnostics, Xiamen, China) respectively by the method of reverse transcriptase polymerase chain reaction(RT-PCR). The details were described in our previous articles [13, 17–19].

### Statistical analysis

Tumor response was evaluated every 2 cycles of chemotherapy according to response evaluation criteria in solid tumors (version 1.1). PFS was defined as the time interval from the first day of treatment to documented disease progression or death of any cause. All of the statistical tests were performed using the SPSS 19.0. Chi-square test or Fisher’s exact test was used to examine the clinicopathologic association of HER2 mutations and response rate comparison. Age differences were compared using the t test for independent samples or the one-way analysis of variance. The Kaplan–Meier method was used to estimate the PFS and the log-rank test was used to analyze PFS between the different groups. Results were considered significantly different if the *p* value was less than 0.05 in a two-way analysis.

## Results

### Patients’ characteristics

**Fig. 1:**
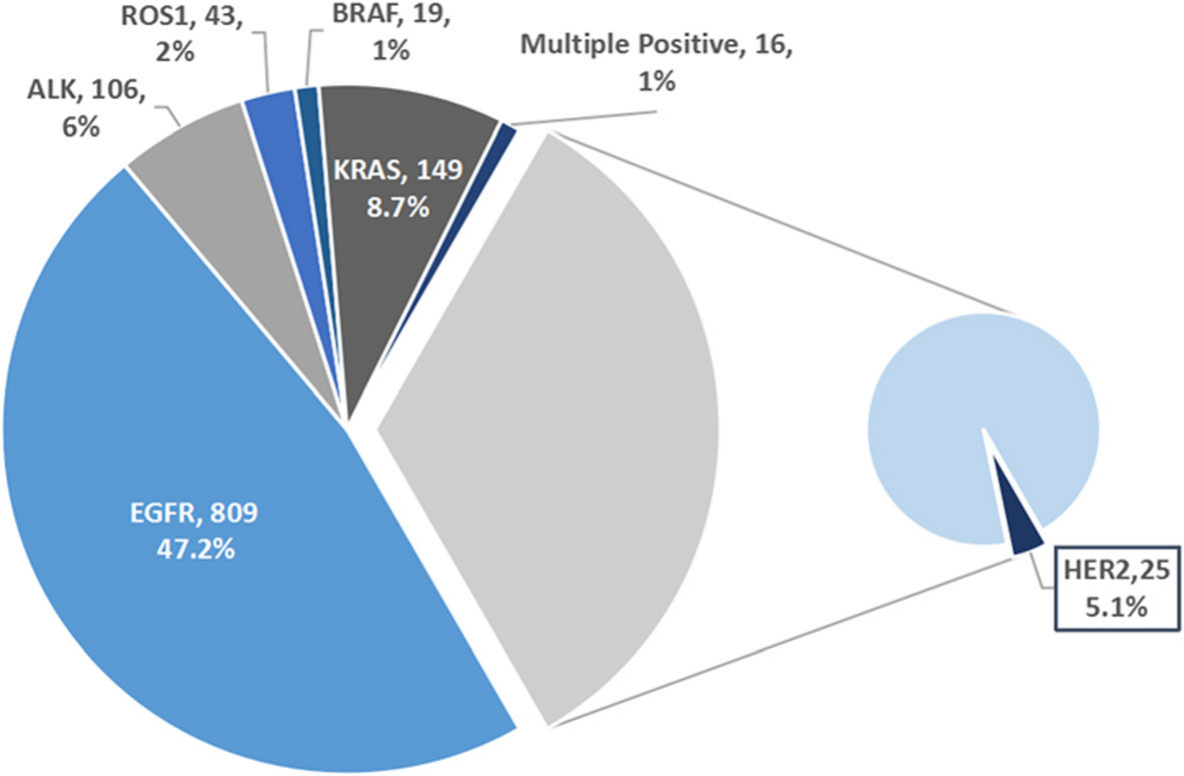
Mutations-testing results of 1714 patients with advanced non-small cell lung cancers.(multiple positive results 7EGFR&ALK, 1EGFR&ROS1, 2EGFR&KRAS, 2EGFR&BRAF, 2ALK&KRAS, 1ROS1&KRAS, 1EGFR&ROS1&KRAS)

From January 2015 to September 2016, a total of 1714 patients with advanced NSCLC underwent testing for *EGFR*, *KRAS*, *BRAF*, *ALK* and *ROS1*.The results showed that there were 809 patients(47.2%) with *EGFR* mutations,149(7.8%) with *KRAS* mutations,19(1.1%) with *BRAF* mutations,106(6.2%) with *ALK* rearrangements, 43(2.5%) with *ROS1* rearrangements, and 16 patients (0.9%) with multiple positive results. In addition, 572 pan-negative patients also have tested their *HER2* status by ARMS and 29 (29/572,5.1%) were identified as *HER2* mutation positive (Fig 1).

*HER2*-mutant lung cancer patients had a median age of 58 (range 44–77 years) and mutations were more common in females (p<0.001),non-smokers (*p* = 0.034) and adenocarcinomas (*p* = 0.002)(Table 1). Twenty-four of 29 patients had available samples for sequencing and had the details variants of *HER2* mutation including 14 with exon20 A775_G776insYVMA, 3with P780_Y781insGSP, 3with G776 > VC, 2with G776 > IC, 1with G776 > LC, and 1with G776C (Additional file 1: Figure S1).

**Table 1:** Clinical characteristics of patients with HER2-mutant lung cancers

Clinical characteristics	Total (*n* = 572)	HER2 negative (*n* = 543)	HER2 positive (*n* = 29)	*P* value
Age, years (median,range)	64(27–92)	64(27–92)	58(44–77)	0.017
< 65	305(53.3%)	283(52.1%)	22(75.9%)	
≥ 65	267(46.7%)	260(47.9%)	7(24.1%)	
Gender				
Male	430(75.2%)	417(76.8%)	13(44.8%)	<0.001
Female	142(24.8%)	126(23.2%)	16(55.2%)	
Smoking status				
Non-smoker	305(53.3%)	284(52.3%)	21(72.4%)	0.034
Smoker	267(46.7%)	259(47.7%)	8(27.6%)	
Histology				
Adenocarcinoma	429(75%)	400(74.0%)	29(100%)	0.002
Non-Adenocarcinoma	143(25%)	117(21.3%)	0	

### Outcomes of chemotherapy: Comparison among oncogenic mutations groups

Patients received first-line pemetrexed-based chemotherapy were eligible for analysis(*n* = 25,14 combined with carboplatin, 7 combined with cisplatin and 4 monotherapy). Since most patients with druggable mutations chose TKI as a first-line treatment, only 74 of 809 *EGFR*-mutant patients and 39 of 149 *ALK*/*ROS1*-rearranged patients were included. While there were a relatively large number of patients with *KRAS* mutation, the first 40 of *KRAS* identified were selected for this study.

The baseline characteristics of patients with *HER2*-mutant were compared with patients with *EGFR*-mutant, *ALK*/*ROS1*-rearranged, and *KRAS*-mutant lung cancers as summarized in Table 2. *HER2*, *EGFR*, *KRAS* mutations and *ALK*, *ROS1* rearrangements did not co-occur with each other in individual patient samples. *KRAS* mutations were more frequently detected in patients with more than 65 years old, male and smokers. And comparison revealed no significant differences in terms of PS score (*p* = 0.269), monotherapy versus combination therapy (*p* = 0.570), maintenance therapy versus non-maintenance therapy(*p* = 0.175).

**Table 2:** Baseline characteristics of patients treated with pemetrexed-containing chemotherapy

Clinical characteristics	HER2	EGFR	ALK/ROS1	KRAS	*P* value
N	25	74	39	40	
Age, years (median,range)	55(44–77)	58(27–80)	54(37–77)	64.5(33–80)	0.002
< 65	21(84.0%)	55(74.3%)	28(71.8%)	20(50.0%)	
≥ 65	4(16.0%)	19(25.7%)	11(28.2%)	20(50.0%)	
Gender					
Male	12(48.0%)	37(50.0%)	20(51.3%)	33(82.5%)	0.004
Female	13(52.0%)	37(50.0%)	19(48.7%)	7(17.5%)	
Smoking status					
Non-smoker	18(72.0%)	56(75.7%)	29(74.4%)	15(37.5%)	<0.001
Smoker	7(28.0%)	18(24.3%)	10(25.6%)	25(62.5%)	
PS					
0–1	22(88.0%)	68(91.9%)	36(92.3%)	32(80.0%)	0.269
≥ 2	3(12.0%)	6(8.1%)	3(7.7%%)	8(20.0%)	
Therapy					
Monotherapy	4(16.0%)	9(12.2%)	3(7.7%)	9(22.5%)	0.570
Plus carboplatin	14(56.0%)	42(56.8%)	24(61.5%)	23(57.5%)	
Plus cisplatin	7(28.0%)	23(31.1%)	12(30.8%)	8(20.0%)	
Maintenance therapy	7(28.0%)	18(24.3%)	13(33.3%)	5(12.5%)	0.175
No maintenance	18(72.0%)	56(75.7%)	26(66.7%)	35(87.5%)	

**Fig. 2:**
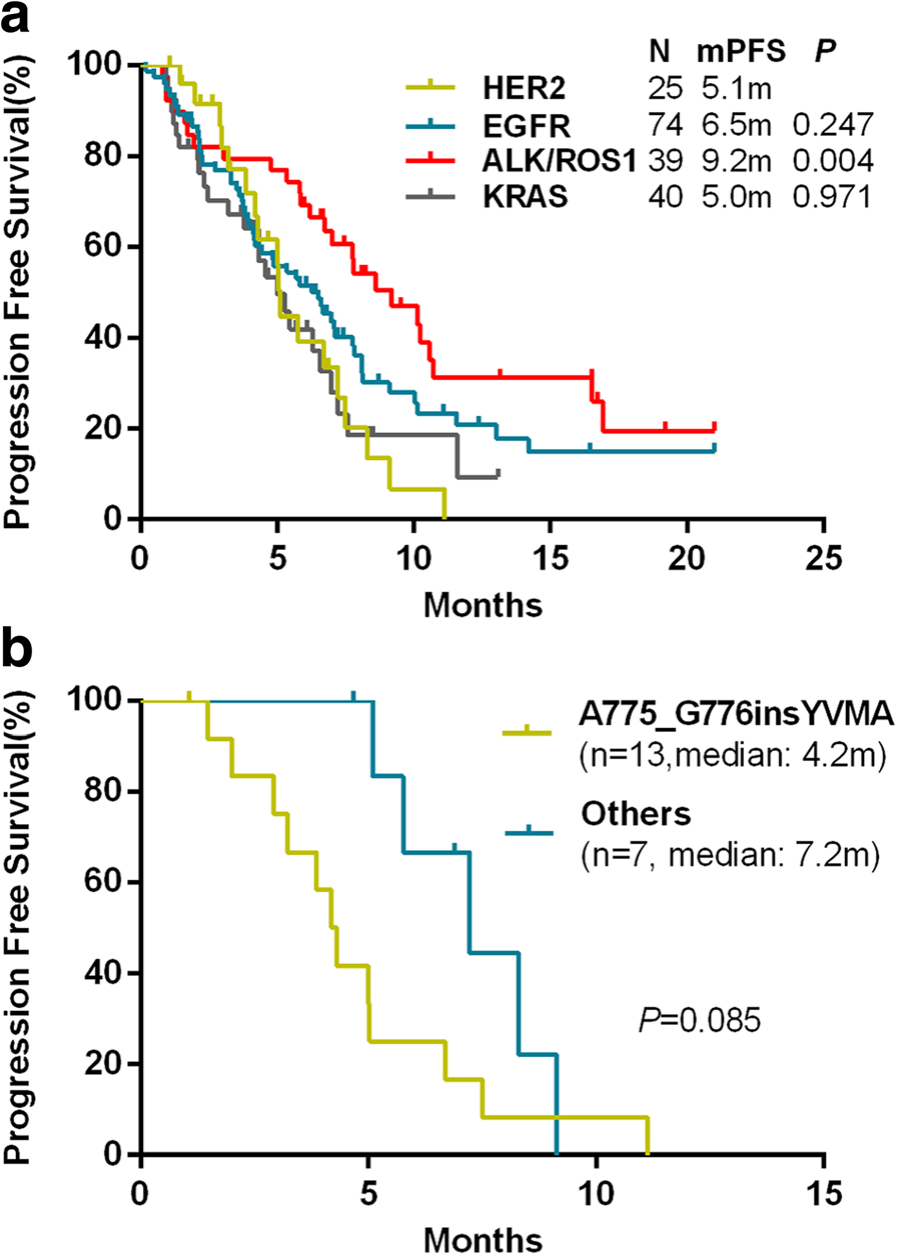
Progression-free survival time. **a**:Progression-free survival time of patients treated with pemetrexed-based therapy The “HER2” group were compared with the “EGFR”, “KRAS” and “ALK/ROS1” groups. **b**: Progression-free survival time of patients with HER2-mutant lung adenocarcinomas treated with pemetrexed-based therapy. The A775_G776insYVMA group were compared with the other variants group (*n*=7, 3with P780_Y781insGSP, 2with G776>IC, 1with G776>LC, and 1with G776C)

The response was evaluated in all 178 patients. Both the objective response rate(ORR) and the disease control rate (DCR) were not significantly different among four groups (Table 3). However, PFS was significantly different among all groups. Patients in the *HER2*-mutant group had a median PFS of 5.1 months (95% confidence interval [CI], 4.90–5.30) (95% CI 4.90–5.30), which was numerically shorter than that of the *EGFR*-mutant group (6.5 months, 95% CI 4.48–8.52, *p* = 0.247) and significantly shorter than that of the *ALK*/*ROS1*-rearranged (9.2 months, 95% CI 6.41–11.99, *p* = 0.004). Similarly, in *KRAS*-mutant lung cancers, PFS (5.0 months, 95% CI 3.67–6.33) was inferior compared with *EGFR*-mutant (6.5 months, *p* = 0.242) and *ALK*/*ROS1*-rearranged (9.2 months, *p* = 0.007) lung cancers. PFS was not significantly different between the *HER2*-mutant and the *KRAS*-mutant lung cancers groups (5.1 vs 5.0 months, *p* = 0.971) (Fig.2a).

**Table 3:** The objective response rate(ORR)and the disease control rate (DCR) of patients treated with pemetrexed-based therapy in four groups

	HER2	EGFR	ALK/ROS1	KRAS	*P* value
n	25	74	39	40	
ORR%	36.0	33.8	41.3	35.0	0.896
DCR%	92.0	78.4	87.2	72.5	0.139

### Outcomes of chemotherapy: Comparison among HER2 variants subgroups

Twenty patients of the 25 patients receiving first-line pemetrexed-based chemotherapy had known HER2 variants (Additional file 1: Figure S1). According to the frequency of the variants, they were divided into the exon20 A775_G776insYVMA group (*n* = 13) and the other variants group (*n* = 7, 3with P780_Y781insGSP, 2with G776 > IC, 1with G776 > LC, and 1with G776C). PFS has a trend to be inferior in the YVMA group, even though no statistically significant difference existed between the 2 groups (4.2 vs 7.2 months, *p* = 0.085) (Fig 2b).

## Discussion

As far as we know, this study is the first study to compare the efficacy of pemetrexed-based chemotherapy between *HER2*-mutant and groups of *EGFR*-mutant, *ALK*/*ROS1*-rearranged and *KRAS*-mutant lung adenocarcinoma. We found that patients with *HER2*-mutant lung cancers had a PFS of 5.1 months that was similar with *KRAS*-mutant (5.0 months, *p* = 0.971) lung cancers, and numerically shorter than *EGFR*-mutant (6.5 months, *p* = 0.247) and significantly shorter than *ALK*/*ROS1*-rearranged (9.2 months, *p* = 0.004) lung cancers, showing that *HER2*-mutant lung cancer patients may have poor outcomes with chemotherapy, which strengthen the importance of developing HER2-targeted drugs in this population. We also investigate the clinicopathologic features in patients with advanced *HER2*-mutant lung adenocarcinomas and found that *HER2* mutations were more common in younger patients, females, non-smokers and adenocarcinomas.

Different from HER2 over-expression and amplification, *HER2* mutations was found to be a distinct entity in patients with NSCLC [20]. *HER2* mutations are found in about 1%–2% of NSCLC [20–22]. In this study, the incidence of *HER2* mutations was 5.1% in *EGFR*/*KRAS*/*BRAF*/*ALK*/*ROS1* negative patients, indicating that *HER2* mutations will be enriched in the population without other driver gene mutations. Consistent with our study, a study from the Memorial Sloan Kettering Cancer Center (MSKCC) group [23] showed that in a selected population with *EGFR*/*KRAS*/*ALK* negative, the incidence of *HER2* mutations can reach up to 6%. In the early stage resection samples, our previous study [6] showed that the presence of HER2 mutations was not correlated with gender, age, or smoking status. However, another retrospective study [24] of resection samples obtained at Fudan University Shanghai Cancer Center found that the incidence of HER2 mutations can reach up to 5.94% in non-smoking patients with lung adenocarcinoma. Similarly, in biopsied samples from advanced NSCLC, our study showed that *HER2* mutations were more common in non-smokers and lung adenocarcinomas. But *HER2* mutations were also frequently detected in younger patients and females in our study. Furthermore, exon20 A775_G776insYVMA was the most frequently alteration.

In the era of targeted therapy, several oncogenic driver mutations were found not only could predict the efficacy of targeted therapy, but also associated with superior outcome of first line pemetrexed chemotherapy, such as *ALK*, *ROS1* and *RET* [12–16]. Thus, we further investigate the association of *HER2* mutation with the efficacy of pemetrexed-based chemotherapy in patients with advanced lung adenocarcinomas. We found that patients with *HER2*-mutant lung cancers had a PFS of 5.1 months. Similar to this study, in the EUHER2 study [25] of patients with *HER2*-mutant lung cancers, ORR and PFS with chemotherapy were 43.5% and 6 months in first-line and 10% and 4.3 months in second-line therapies. Our study also showed that *HER2*-mutant lung cancers had a similar PFS of pemetrexed-based chemotherapy with *KRAS*-mutant lung cancers (5.0 months), which was inferior compared with *EGFR*-mutant(6.5 months) and *ALK*/*ROS1*-rearranged (9.2 months), indicating that *HER2* mutation might predict a poor efficacy of pemetrexed-based chemotherapy, just like *KRAS* mutation. Although pemetrexed-based chemotherapy had the longest duration among chemotherapies(pemetrexed/taxane/gemcitabine/vinorelbine/etoposide±platinum) for patients with *HER2* mutations according to Eng et al’s study [26] and Gow et al’s study [27], outcomes of pemetrexed for *HER2* were poor compared to other oncogene subgroups,such as *ALK* and *ROS1*. Furthermore, we further divide *HER2* mutations into the exon20 A775_G776insYVMA group and the other variants group and it was the first time that we found that patients with YVMA insertion were associated with an inferior PFS (4.2 vs 7.2 months, *p* = 0.085).

Currently, NCCN guideline recommend trastuzumab and afatinib as the targeted therapeutic options for patients with advanced *HER2*-mutant NSCLC. While, in EUHER2 study [25], afatinib showed a modest response of 18.2% and median PFS of 3.9 months even though this drug has showed response in all 3 assessable patients with *HER2*-mutant adenocarcinoma in a preliminary study [28]. Meanwhile, several other studies [5, 7, 8] investigated the efficacy of other irreversible pan-HER receptor family inhibitors, dacomitinib, neratinib, or neratinib combining with mTOR inhibitors in advanced NSCLC patients harboring *HER2* mutations and showed a moderate response of 12%–21%. Although these ORR or PFS are much diminished compared with those of TKIs directed at other targets in NSCLC, HER2-targeted drugs is still promising. A phase II study recently investigated a novel EGFR/HER2 inhibitor, pyrotinib, in heavily pre-treated patients with *HER2*-mutant adenocarcinomas and found a promising results with RR of 54.5%(6/11) and median PFS of 6.2 months [9]. Large number cohort study is still needed to validate the efficacy of pyrotinib in this setting.

Our study does have several limitations. First, it was a retrospective study with limited patients number(*n* = 25), while this study presented the real world nature in Chinese population. Second, *HER2* mutation testing was performed using the method of ARMS, thus some rare mutations might be missed in our population. Next generation sequencing (NGS), which allows for simultaneous testing for multiple mutations using one platform and one sample, is emerging as an important method for identification of gene mutations in NSCLC, but single-gene sequencing is still more widely used. Thirdly, a substantial part of the patients with *HER2* mutations also participant into the previous clinical trial of HER2-targeted drugs [9], thus the overall survival might be heavily influenced by the subsequent therapy.

## Conclusions

In conclusion, *HER2* mutations were more frequent happened in younger patients, females, non-smokers and adenocarcinomas of advanced NSCLC. Patients with *HER2*-mutant lung adenocarcinomas, especially YVMA insertion, showed poor response to pemetrexed-based chemotherapy. Thus, developing HER2-targeted drugs to improve their poor prognosis is urgently needed for this population.

## Additional file

Additional file 1: **Figure S1.** Study flow chart. (PDF 160 kb)

## Abbreviations

ARMS: Amplification refractory mutation system
HER2: Human epidermal growth factor receptor2
NCCN: The National Comprehensive Cancer Network.
NSCLC: Non-small cell lung cancers
ORR: The objective response rate
PFS: Progression free survival
RT-PCR: Reverse transcriptase polymerase chain reaction
