# Supplementary figures and images for: Outcomes of Pemetrexed-based chemotherapies in HER2-mutant lung cancers
Source: BMC Cancer. 2018 Mar 27;18:326. doi: 10.1186/s12885-018-4277-x (PMC5869778; doi:10.1186/s12885-018-4277-x)

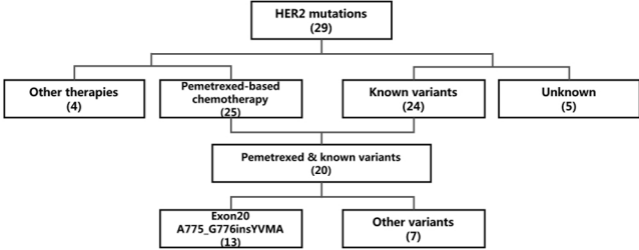

Supplement: Supplementary file 1 — Figure S1. Study flow chart. (PDF 160 kb) [file 12885_2018_4277_MOESM1_ESM.pdf]
